# Hemodynamic support during catheter ablation of ventricular arrhythmias in patients with cardiogenic shock

**DOI:** 10.3389/fcvm.2023.1145123

**Published:** 2023-04-27

**Authors:** Michael Spartalis, David Zweiker, Eleftherios Spartalis, Dimitrios C. Iliopoulos, Gerasimos Siasos

**Affiliations:** ^1^ 3rd Department of Cardiology, Sotiria Thoracic Diseases General Hospital, National and Kapodistrian University of Athens, Athens, Greece; ^2^ Department of Cardiology, Medical University of Graz, Graz, Austria; ^3^ Laboratory of Experimental Surgery and Surgical Research “N. S. Christeas”, National and Kapodistrian University of Athens, Medical School, Athens, Greece

**Keywords:** hemodynamic, catheter ablation, ventricular arrhythmia, cardiogenic shock, electrical storm

## 1. Introduction

Cardiogenic shock is characterized by systemic hypoperfusion of tissues as a result of a reduced cardiac output despite sufficient left ventricular filling pressure and circulatory volume (1–4). Cardiopulmonary support must be provided as promptly as feasible in this scenario, especially when individuals do not respond to drug therapy (1–4). Prompt commencement of cardiopulmonary support could mitigate the effects of systemic hypoperfusion, such as deteriorating ischemia and diminishing heart function (1–4). Several investigations have published evidence on individuals with cardiogenic shock due to ventricular arrhythmias (1–3). However, the majority of these studies do not include a thorough assessment of rhythm disturbance treatment outside hemodynamic stability with cardiopulmonary support (1–3). Most investigations indicate that rhythm disturbances are a reversible trigger of cardiac arrest, with patients' prognoses solely dependent on rhythm stability (3, 4). In rare instances, sinus rhythm restoration may occur immediately after achieving hemodynamic stabilization (3, 4). When refractory rhythm disturbances hinder clinical stability and weaning from cardiopulmonary support, catheter ablation may be used to restore sinus rhythm (1–3).

Catheter ablation is a viable therapy for ventricular tachycardia (1–3). It is linked with a decrease in ventricular tachycardia burden, an improvement in quality of life, and an increase in longevity in certain individuals (1–3). Ablation of ventricular arrhythmias has a synergistic function with cardiopulmonary support since the two combined constitute a realistic and successful approach for achieving electrical and hemodynamic stability (1–3).

Considering advancements in clinical therapy and methods for cardiopulmonary support, cardiogenic shock and electrical storm are primary causes of death and morbidity in heart failure individuals (4–8). In particular, cardiogenic shock caused by refractory ventricular arrhythmia bears a mortality incidence of up to fifty percent (9).

Individuals with cardiogenic shock are prone to ventricular arrhythmias, which, despite the administration of effective antiarrhythmic medications, may compromise or prevent successful weaning off cardiopulmonary support (1, 3). The treatment of this high-risk category of individuals should be confined to either rescue ablation of ventricular arrhythmia or heart transplant due to its severe difficulty (1). This could be exacerbated by short- and long-term mortality and donor supply constraints (1). Moreover, in the presence of hemodynamic impairment, ventricular tachycardia ablation becomes more complicated, impeding the ability to preserve the rhythm disturbance lengthy enough for accurate mapping and ablation without further hemodynamic instability and cerebral desaturation (3).

## 2. Catheter ablation and cardiopulmonary support in cardiogenic shock

Despite cardiopulmonary support, many ventricular arrhythmias are poorly tolerated hemodynamically, which might be due to a decrease in right ventricle output or inadequate hemodynamic left ventricle support in relation to tachycardia cycle durations and the degree of left ventricular failure (3). However, the availability of cardiopulmonary support at the onset of ablation may enhance the acute procedural results, as demonstrated by previous investigations of ventricular tachycardia ablation with hemodynamic support (10–14).

The treatment of cardiogenic shock continues to be arduous, with a substantial fatality rate (3). This is consistent regardless of whether the cardiogenic shock is a result of decompensation of heart failure, acute myocardial infarction, or refractory ventricular arrhythmia (15–19).

The higher mortality and morbidity documented in cardiogenic shock individuals indicate their underlying severe condition (3). The assertion of worsening cardiogenic shock as the primary lead of mortality gives credence to this further (3). In particular, individuals who pass away while receiving care at the index hospital are more prone to have a reduced ejection fraction and to be in cardiogenic shock for a prolonged duration prior to and following the ablation (1). Nevertheless, it appears ambiguous if fatalities are associated with extended durations in the cardiogenic shock prior to ablation and whether earlier treatment would have resulted in higher life expectancies at discharge (1, 3).

In addition, a number of investigations have demonstrated that patients who originally displayed cardiogenic shock demanding cardiopulmonary support and afterward formed ventricular tachycardia possess a greater in-hospital fatality rate than patient populations who at first needed hemodynamic support for both cardiogenic shock and ventricular tachycardia (1, 3).

In certain circumstances, the rationale for extremely early ablation could be to regulate hemodynamics with the intention of decreasing the impact of arrhythmia (1). During ablation cases, additional hemodynamic decompensation might be noted in normally hemodynamically stable individuals (1). In a contemporary investigation, the clinical results of individuals with electrical storm who have abrupt periprocedural hemodynamic decompensation after ablation treatments were evaluated (20). The research demonstrated extremely robust acute success rates but 62% in-hospital death rates (20). Conversely, when administered as rescue therapy, ablation was still correlated with survival to discharge for the majority of individuals (20).

A few individuals who are released from the hospital could experience ventricular tachycardia reappearance under the monitoring threshold of their implanted cardioverter-defibrillator (ICD) (1).

In these contexts, clinical management is challenging and must consider comorbidities and baseline cerebral and cognitive state.

## 3. Discussion

In individuals receiving catheter ablation of ventricular tachycardia with complex substrates, several comorbidities, and a current record of repeated ventricular tachycardia-related ICD shocks, the initiation of cardiopulmonary support is crucial (1). These individuals have an inherent risk of periprocedural mortality that increases in proportion to the rhythm disturbance load (1). The delayed commencement of cardiopulmonary support following procedure-related acute hemodynamic decompensation has a significant influence on prognosis, with up to 50% death at 21 ± 7 months of follow-up (21).

The Milan cohort of 74 extracorporeal membrane oxygenation (ECMO)-supported ablation treatments revealed equivalent outcomes. In addition, faster ventricular tachycardias were found in the five patients in whom ECMO was used as an intra-procedure rescue method, lowering the effectiveness of ECMO in avoiding end-organ hypoperfusion (11).

The University of Pennsylvania described a cohort of 21 individuals undergoing ablation of ventricular tachycardia who needed ECMO support due to severe cardiac damage (20). Even with an 83% overall success rate, 88% of individuals deceased after a mean follow-up of 10 days, 2% of them due to refractory ventricular tachycardia, indicating that ECMO support as a rescue approach permits ablation but cannot avoid immediate death attributable to cardiac decompensation (20).

Nonetheless, the outcomes of ablation with preemptive support (Impella or Tandem Heart) are encouraging. The direct contrast of rescue vs. preemptive ventricular assist device (VAD) vs. the lack of cardiopulmonary support throughout ablation of ventricular tachycardia in a population of 21 individuals revealed a higher 30-day fatality rate in the rescue cluster, with a longer procedure time, apart from comparable ablation outcomes (22). Consequently, the detection of individuals who require preventative cardiopulmonary support is critical and must be established on a clinical examination performed upon hospitalization. The expertise of large volume centers has favored the development of tools that, based on clinical arrhythmia profile, hemodynamic tolerability, medical state, and comorbidities, detect potential individuals at a greater danger of unfavorable periprocedural events (1).

On the basis of clinical factors at hospitalization, efficient risk classification of newly admitted individuals is possible (21). Six demographic, clinical, and procedure-related factors were investigated as possible predictive variables in a large multicenter study of 1,251 individuals adopting the Survival Tree assessment approach. Left ventricular ejection fraction (LVEF), ICD/cardiac resynchronization device, and prior ablation were recognized as the strongest indicators of arrhythmia recurrence; LVEF, previous ablation, and electrical storm were indicated as the greatest indicators of death. There were three distinct categories with considerably varying survival probabilities. The high-risk category had the greatest incidence of death and arrhythmia recurrence (23).

The PAINESD risk score was created by a team from the University of Pennsylvania (21). Age, ischemic cardiomyopathy, lower LVEF, diabetes, chronic obstructive pulmonary disease, electrical storm, and New York Heart Association functional class III/IV were incorporated in the PAINESD risk score as markers of abrupt cardiac decline. Additionally, the administration of general anesthesia was shown to be related to an elevated risk of acute cardiac failure (21).

In a cohort of 75 individuals receiving scar-related ventricular tachycardia ablation, preemptive VAD placement directed by the PAINESD scoring system was helpful, yielding greater benefits than a propensity-matched cohort of 75 individuals receiving ablation absent VAD placement (10). Despite equal ablation outcomes, the population who received ablation with cardiopulmonary support had greater longevity, transplant-free survival, and a decreased risk of acute cardiac decompensation following ablation than the population who did not get cardiopulmonary support (10).

These findings suggest that ventricular tachycardia individuals must be accurately risk-stratified prior to the procedure (1). Even when approaching ablation in an evident stable cardiopulmonary status, the majority of complex individuals may subsequently encounter periprocedural abrupt hemodynamic deterioration, which has a significant influence on subjects' mortality (1). In the lack of contraindications, individuals at high risk for ablation must be safeguarded with prophylactic cardiopulmonary support regardless of the pathophysiology of the cardiovascular disorder, the method devised for ablation, and their hemodynamic condition at the start of the operation (1).

When a bailout catheter ablation technique is required to address recalcitrant rhythm disturbances, cardiopulmonary support is an efficient therapy for cardiogenic shock caused by recalcitrant ventricular arrhythmias (1–3). Preventative assistance for high-risk individuals requiring ablation of unstable ventricular arrhythmias is also beneficial (1). The device must be chosen based on team skills and the availability of tertiary referral centers (1, 3). The goals of cardiopulmonary support must be to restore an acceptable circulatory state, facilitate catheter ablation, and bridge individuals to target therapy for cardiac failure.

## Author contributions

MS: wrote the first draft of the report. DZ, ES, DI, and GS: helped to wrote the final version. All authors contributed to the article and approved the submitted version.

## Conflict of interest

The authors declare that the research was conducted in the absence of any commercial or financial relationships that could be construed as a potential conflict of interest.
